# Effect of Allopregnanolone on Spatial Memory and Synaptic Proteins in Animal Model of Metabolic Syndrome

**DOI:** 10.3390/brainsci11050644

**Published:** 2021-05-15

**Authors:** Shaimaa Nasr Amin, Shaimaa Abdalaleem Abdalgeleel, Mubarak Ali Algahtany, Sherif Ahmed Shaltout, Walaa Bayoumie El Gazzar, Dalia Azmy Elberry

**Affiliations:** 1 Department of Basic Medical Sciences, Faculty of Medicine, Hashemite University, Zarqa 13133, Jordan; Sherif@hu.edu.jo (S.A.S.); wallagazzar@hu.edu.jo (W.B.E.G.); 2 Department of Medical Physiology, Faculty of Medicine, Cairo University, Cairo 11451, Egypt; dalia_azmy@cu.edu.eg; 3 Department of Biostatistics and Epidemiology, National Cancer Institute, Cairo University, Cairo 11451, Egypt; 4 Division of Neurosurgery, Department of Surgery, College of Medicine, King Khalid University, Abha 61421, Saudi Arabia; Mbalgahtany@kku.edu.sa; 5 Department of Pharmacology, Faculty of Medicine, Benha University, Benha 13511, Egypt; 6 Department of Biochemistry, Faculty of Medicine, Benha University, Benha 13511, Egypt

**Keywords:** metabolic syndrome, spatial memory, synaptic plasticity, synaptophysin, GA*P*-43, allopregnanolone

## Abstract

Metabolic Syndrome (MetS) is considered a common disorder, especially with a sedentary lifestyle and unhealthy food consumption. Cognitive impairment is one of the MetS consequences that worsens the quality of life of the patients. The study aimed to assess the therapeutic effect of the neurosteroid Allopregnalonone on spatial memory and, therefore, the expression of two synaptic plasticity markers in the hippocampus. Thirty-two male rats were divided into four groups: control groups, MetS, and MetS + Allopregnalone. Spatial memory has been evaluated by the Y-maze task and blood pressure measured by the rat tail method. Biochemical evaluation of serum glucose, insulin, lipid profile, and hippocampal expression of Synaptophysin and Associated Protein 43 (GA*P*-43) were performed for assessing Allopregnanolone on serum and hippocampal markers. Allopregnanolone therapy improved working spatial memory, hypertension, and biochemical markers measured in the serum and hippocampus.

## 1. Introduction

Metabolic syndrome (MetS) is a common metabolic disorder correlated to a sedentary lifestyle and junk food consumption. The syndrome manifestations are abnormal lipid profile, hypertension, hyperglycemia, insulin resistance (IR), and abdominal obesity [1].

Cognitive dysfunction and dementia are known comorbidities associated with MetS. The suggested mechanisms for impaired cognition in MetS may be related to oxidative stress, abnormal metabolism, inflammation, and vascular reactivity abnormalities. These abnormalities, in turn, disturb the homeostasis of the internal environment around the neurons under basal conditions and more impairment during activity [2]. As encountered in the metabolic syndrome, insulin resistance is found to reduce neurosteroids [3], and neurosteroids administration has been found to improve hypertension in obesity [4].

Neurosteroids are steroid hormones synthesized de novo in the brain and peripheral nervous tissues [5]. Allopregnanolone is an essential neurosteroid in the brain, synthesized locally or in the periphery, and reaches the brain by crossing the blood-brain barrier [6]. Allopregnanolone levels were reported to be downregulated in neurodegenerative diseases [7]. 

The current work aimed to evaluate the impact of the neurosteroid Allopregnanolone administration as a modulator for Gamma-Amino-Butyric Acid (GABA)-A receptors on working spatial memory and hippocampal expression of Synaptophysin and Associated Protein 43 (GAP 43) expression in an animal model of metabolic syndrome (induced by high fructose diet for eight weeks).

## 2. Materials and Methods

### 2.1. Experimental Animals

Thirty-two male Sprague–Dawley rats weighing 100–150 g, eight weeks old, were used in this study. Rats were obtained and housed in the ophthalmology research institute, Giza, Egypt. The animals were housed in stainless steel cages (4 rats/cage to avoid isolation stress) at the temperature range of 22 ± 2 °C, under a 12:12 light/dark cycle. The bedding in the cages was wood chips. Rats were allowed to acclimatize for ten days before experimentation; adequate water and chow were available ad libitum.

### 2.2. Induction of Metabolic Syndrome

Induction of metabolic syndrome in the albino rat model will be achieved by administration of a high-fructose diet (HFD) for eight weeks. HFD is found to induce insulin resistance in rats 3–4 weeks after consuming 60% HFD daily [8]. High-fructose diet (HFD) composed of: starch 2%, sucrose 2%, fructose 60.0%, protein 20.0%, fat, 5.0%, vitamins 1.0%, minerals 5.0% [9]. This formula was mixed with a normal diet in a ratio of 6:4; thus, rats received 60% HFD daily. 

### 2.3. Experimental Design

Rats were divided into the following groups (8 rats/group): 

*Group I*- *Negative control:* consumed ad libitum water and standard chow diet (7% simple sugars, 3% fat, 50% polysaccharide, 15% protein), energy 3.5 kcal/g [10], and received the vehicle for Allopregnanolone.

*Group II: Allopregnanolone group (Positive control):* Consumed ad libitum water and standard chow diet (7% simple sugars, 3% fat, 50% polysaccharide, 15% protein), energy 3.5 kcal/g [11] and received Allopregnanolone (Sigma Aldrich, St. Louis, MO, USA) (20 mg/kg, orally) for eight weeks. Allopregnanolone initially dissolved in absolute ethanol then diluted with saline, yielding a final concentration of 20 mg/mL of administration solution. The percentage of ethanol and saline in the final volume is 20% and 80%, respectively [12].

*Group III: Metabolic Syndrome (MetS):* consumed ad libitum water and HFD for 8 weeks (starch 2%, sucrose 2%, fructose 60.0%, protein 20.0%, fat, 5.0%, vitamins 1.0%, minerals 5.0%) [9]. This formula was mixed with a normal diet in a ratio of 6:4; thus, rats received 60% HFD daily.

*Group IV: MetS + Allopregnanolone group:* received diet as described in group III and starting from week three till the end of week 8; Allopregnanolone administered as described in group II.

Randomization was performed while selecting the rats for each group. For groups, no randomization was done; all rats within the same group received the same protocol for therapy and measurements. At the time of testing, rats were seventeen weeks old. The Y-maze test evaluated rats’ spatial working memory, and on the second-day rats, blood pressure was measured (the same day of euthanasia and sampling).

Beginning → 10 days acclimatization → 8 weeks of modeling → Y-maze test → after one day of Y-maze testing, blood pressure evaluated followed by euthanasia and sampling.

### 2.4. Assessment of Spatial Working Memory

#### 2.4.1. Y-Maze Task

Spontaneous alternation in rats refers to rodents’ natural tendency to spontaneously choose alternate arms in a Y-maze, and it is considered a quick and relatively simple test of spatial working memory [7].

#### 2.4.2. Apparatus

A wooden Y-maze has three equal size arms (60 cm long, 11.5 cm wide, and 25 cm high). Rats placed in the center of the apparatus to explore the maze for 8 min. 

Parameters measured are the number of arm entries, the number of alternations (manually recorded), and the alternation score. Any three consecutive choices of three different arms were counted as a correct choice or alternation. The alternation score is a calculation that reflects the spatial working memory using the number of correct choices or alternation and the total number of arm entries as follow:

Alternation score (%) = (number of alternations)/(total arm entries − 2) × 100 [7].

Tests were performed during the light phase of the light/dark cycle after 30 min of acclimatization. Blinding was used during the Y-maze test and analysis of the videos as the investigator performed the tests and the analysis provided with the animals as serial numbers without declaring the groups or therapies. The maze was cleaned with 20% ethanol, and we waited till its odor evaporated before placing another animal to avoid the effect of any olfactory cues.

### 2.5. In Vivo Measurement of the Arterial Blood Pressure

The blood pressure of animals was indirectly measured by a non-invasive blood pressure monitor (LE 5001, LETICA scientific Instruments, Espania) from conscious rats’ tail by the tail-cuff technique. In the tail-cuff technique, animals are warmed for 30 min at 28 °C in a thermostatically controlled heating cabinet (Ugo Basille, Italy) to detect tail artery pulse better. The tail will be passed through a miniaturized cuff, and a tail-cuff sensor connected to an amplifier (LE 5001, LETICA Scientific Instruments, Espania) and BP will be recorded on a chart [13].

At the end of the work, blood samples were collected from retroorbital venous sinuses to measure serum glucose, insulin, and lipid profile. Animals were euthanized, then brains were extracted and dissected at the level of the hippocampus for assessment of the expression of Synaptophysin and Growth Associated Protein 43 (GA*P*-43):

### 2.6. Biochemical Measurements

Serum glucose was performed according to the method of Passing and Bablok [14], 1983. The kits were supplied by Diamond Diagnostics Inc., Holliston, MA, USA.

Serum insulin measured by enzyme-linked immunosorbent assay (ELISA) kits (DRG, USA) according to manufacturer’s instructions.

Homeostasis model assessment of insulin resistance (HOMA-IR):

HOMA-IR was calculated as fasting insulin (U/L) × fasting glucose (mg/dL)/405, as described by Matthews et al. [15].

HOMA value of more than 4.0 is an index for insulin resistance [16].

Serum cholesterol measured according to the method of Allain et al. [17]. Serum triglycerides were performed according to the method of Glick et al. [18]. Serum HDL-cholesterol was performed according to the method of Lopez-Virella et al. [19].

### 2.7. Gene Expression by Real-Time PCR of Synaptophysin and Growth Associated Protein 43 (GAP-43)

Total RNA extraction:

According to manufacturer instructions, total RNA was extracted from hippocampal homogenate using RNA Isolation System (Qiagen, Germantown, MD, USA). The RNA concentrations and purity were measured with an ultraviolet spectrophotometer.

*Complementary DNA (cDNA) synthesis:* The cDNA was synthesized from 1 μg RNA using SuperScript III First-Strand Synthesis System described in the manufacturer’s protocol (#K1621, Fermentas, Waltham, MA, USA). 

*Real*-*time quantitative PCR*: Real-time PCR amplification and analysis were performed using an Applied Biosystem with software version 3.1 (StepOne™, Foster City, CA, USA). The reaction contained SYBR Green Master Mix (Applied Biosystems), gene-specific primer pairs, which were shown in Table 1 and designed with Gene Runner Software (Hasting Software, Inc., Hasting, NY, USA) from RNA sequences from the gene bank. All primer sets had a calculated annealing temperature of 60°. Quantitative RT-PCR was performed in a 25-μL reaction volume consisting of 2X SYBR Green PCR Master Mix (Applied Biosystems), 900 nM of each primer, and 2 μL of cDNA. Amplification conditions were: 2 min at 50°, 10 min at 95°, and 40 cycles of denaturation for 15 s, and annealing/extension at 60° for 10 min. Data from real-time assays were calculated using the v1·7 sequence detection software from PE Biosystems (Foster City, CA, USA). Relative expression of studied gene mRNA was calculated using the comparative Ct method. All values were normalized to glyceraldehyde-3-phosphate dehydrogenase, used as the control housekeeping gene, and reported as fold change over background levels detected in the diseased groups.

### 2.8. Statistical Methods

The Statistical Package of Social Science (SPSS) (version 26) was used to generate results. The normality of the data was tested using the Kolmogorov–Smirnov single-sample test. As the data were normally distributed, they were presented as mean and standard deviation (SD). For comparison, an ANOVA test was used to compare groups. Post hoc multiple comparisons were made using the Tukey test. Pearson correlation was used to correlate continuous data. A (*p* ≤ 0.05) was considered significant.

## 3. Results

As shown in Table 2:

Mean serum glucoses: it was significantly higher in MetS than in negative control for Allopregnanolone, positive control for Allopregnanolone and MetS + Allopregnanolone (*p*-value ≤ 0.001, < 0.001 and 0.005 respectively).

In addition, it is significantly lower in MetS+ Allopregnanolone than in negative control for Allopregnanolone and positive control for Allopregnanolone (*p*-value < 0.001).

Mean serum insulin: it was significantly lower in negative control for Allopregnanolone than other groups (*p*-value < 0.001), while it was significantly higher in the MetS group than positive control for Allopregnanolone and MetS+ Allopregnanolone (*p*-value < 0.001 and 0.002 respectively).

Mean HOMA-IR: it was significantly higher in MetS than in other groups ((*p*-value < 0.001), and it was significantly higher in MetS + Allopregnanolone than negative control for Allopregnanolone and positive control for Allopregnanolone (*p*-value < 0.001).

Mean serum Triglycerides: it was significantly higher in MetS than negative control for Allopregnanolone and positive control for Allopregnanolone (*p*-value < 0.001), and it was significantly higher in MetS + Allopregnanolone than negative control for Allopregnanolone and positive control for Allopregnanolone (*p*-value < 0.001; 0.001 respectively)

Mean serum Cholesterol: it was significantly higher in MetS than in negative control for Allopregnanolone, positive control for Allopregnanolone and MetS + Allopregnanolone (*p*-value = < 0.001, < 0.001 and 0.005 respectively).

Mean Serum HDL: it was significantly lower in MetS than in other groups (*p*-value < 0.001), and it significantly lower in MetS + Allopregnanolone than in other groups (*p*-value < 0.001).

Mean Hippocampal Synaptophysin: All groups were significant from each other, with the lowest mean in the MetS group (*p*-value < 0.001).

Mean Hippocampal Growth Associated Protein 43 (GAP43) (relative expression): it was significantly higher MetS than other groups (*p*-value < 0.001), the mean higher in MetS + Allopregnanolone than in positive control for Allopregnanolone (*p*-value < 0.001).

Table 3 shows:

Mean systolic blood pressure: it was significantly higher in MetS than in other groups (*p*-value < 0.001), and it was significantly higher in MetS + Allopregnanolonen than in positive control for Allopregnanolone (*p* value = 0.002)

Mean diastolic blood pressure was significantly higher in the MetS group than in other groups (*p*-value < 0.001).

Mean arterial blood pressure: significantly higher in the MetS group than in other groups (*p*-value < 0.001).

**Figure 1:**
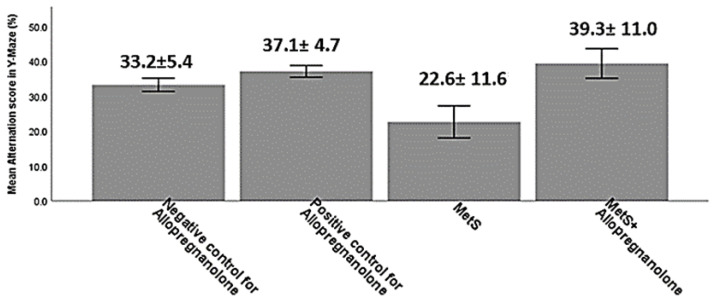
Alteration score in the studied groups in the Y-maze test. Alternation score (%) = (number of alternations)/(total arm entries − 2) × 100. Data are expressed as mean ± SD; *n* = 8 in each group.

As shown in Figure 1:

Alternation score in Y-Maze (%), negative control for Allopregnanolone (33.2 ± 5.4), positive control for Allopregnanolone 37.1 ± 4.7, MetS 22.6 ± 11.6 and for MetS+ Allopregnanolone (39.3 ± 11.0) with a significant lower mean in MetS group than positive control for Allopregnanolone and for MetS + Allopregnanolone (*p*-value = 0.031 and 0.009 respectively).

**Figure 2:**
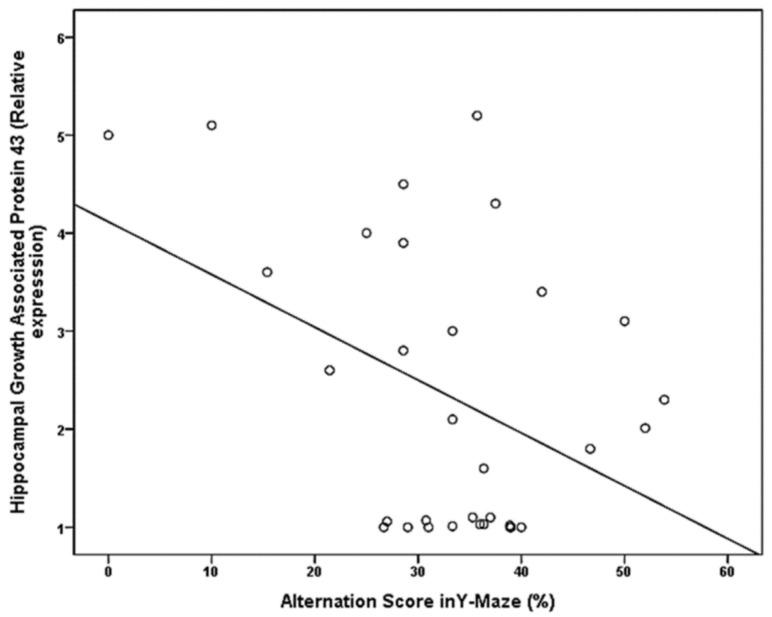
Significant negative correlation between the alternation score and Hippocampal Growth Associated Protein 43 expression.

**Figure 3:**
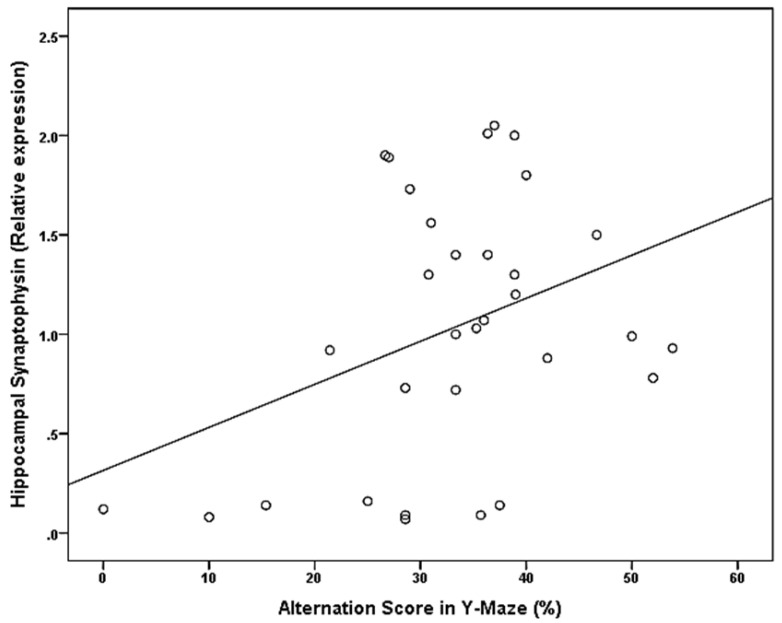
Significant positive correlation between alternation score and hippocampal synaptophysin expression.

Table 4 and Figure 2 and Figure 3 show:

Significant positive correlation between alternation score and hippocampal synaptophysin expression (*p*-value = 0.039), Significant negative correlation between the alternation score and Hippocampal GAP 43 expression (*p*-value = 0.019), however no significant correlation was found between serum glucose and serum insulin, serum triglycerides, serum cholesterol, serum HDL and alternation score (*p*-value = 0.640, 0.062, 0.094, 0.262 and 0.077 respectively).

## 4. Discussion

MetS is a critical universal disorder that affects multiple brain regions, especially the hippocampus, concerned with memory and learning processes [20,21]. A commonly used maze test is the Y-maze task [22], used in our study to assess working spatial memory via calculating the alternation score. Our results revealed marked deterioration of spatial working memory in the form of a significant (*p* ≤ 0.05) decrease in the alternation score of the MetS group compared to the negative control group (Figure 1). These results concord with previous studies that revealed a significant defect in spatial working memory processes in rodents with diet-induced MetS, using the Barnes maze [23], the Morris water maze [24], and novel objects test (NORT) [20].

The present study showed a significant decrease in the relative expression of hippocampal Synaptophysin of MetS (Table 2). This effect most probably was a reason for the apparent spatial working memory changes in the MetS group, evidenced by the significant positive correlation between the alternation score and relative expression of Synaptophysin (Figure 3). This outcome was consistent with the work done by Trevino et al. [20], who claimed that immunoreactivity to Synaptophysin in the hippocampus of high-calorie diet-induced MetS animals (with memory impairments) compared to control. Synaptophysin is an essential marker of neuronal plasticity [25]. It is an abundant integral membrane protein of presynaptic vesicles involved in regulating neurotransmitter release and synaptic plasticity [26]. Synaptophysin plays a crucial role in neurogenesis and neuronal interaction, particularly in degenerative diseases such as Alzheimer’s disease [27].

Astrocytes play a vital role in maintaining neurotransmission and maintaining synapses, reflecting cognitive function [28]. Among various messengers released by astrocytes, Thrombospondin-1 (TS*P*-1) [26]. TS*P*-1 deficiency impairs the presynaptic proteins, such as Synaptophysin [29]. Moreover, activation of Toll-like receptor 9 (TLR9) by hyperglycemia mediates oxidative stress and astrocytic dysfunction, leading to reduced TS*P*-1 secretion and synaptophysin expression [30]. Since the metabolic disorders in MetS (dyslipidemia, hyperglycemia, and IR); were all demonstrated in our results; this may suggest excessive oxidative stress and inflammation, increasing the susceptibility of the CNS to injury [31].

Furthermore, fructose may directly affect brain functions and increase hippocampal and cortical glucose transporter 5 (GLUT5) [32]. Fructose has higher reactivity than glucose, producing several alterations and increasing oxidative stress events [33].

GA*P*-43, as with Synaptophysin, is expressed in the terminals of presynaptic vesicles [34]. It worth noting that GA*P*-43 is involved in synaptic plasticity processes such as long-term potentiation (LTP) and is correlated to anatomical arborization of new synapses [35]. Unexpectedly and contradictory to many previous studies, our results revealed a significant increase in the relative expression of hippocampal GAP 43 in the MetS group compared to the control group (Table 2).

Additionally, there is a significant negative correlation between alternation score and relative expression of GAP 43 (Table 4, Figure 2). These findings are not concomitant with Gu et al., [36] research which demonstrated that streptozotocin (STZ)-induced diabetes in rat models produced marked deterioration of spatial learning and memory with downregulation of GA*P*-43 expression in the hippocampus. This discrepancy in results might be due to the diversity of the animal models employed [36]. However, our results agree with the work done by Sandelius et al. [37].

Besides, increased GA*P*-43 in the area of infarction in experimental brain ischemia has been reported indicating neuronal injury and possible regeneration [38,39]. These previous studies may partly explain our results, as it was documented that fructose administration leads to a reduction in cerebral blood flow (CBF) in the hippocampus and other parts of the brain [40,41]. Moreover, Hung et al. [42] reported that lipopolysaccharide (LPS) induced GA*P*-43 through ROS and nuclear factor-B (NF-B) [42]. This fact may be linked to ROS accumulation with high fructose intake [43].

Our results demonstrated the prominent therapeutic effect of Allopregnanolone on working spatial memory, evidenced by the significant improvement of alternation score in the Allopregnanolone group compared to the MetS group (Figure 1). These results are concomitant with the study done by Park et al., [6], who assessed the effect of Allopregnanolone on spatial learning and memory in aged mice using the radial arm water maze. Additionally, Frye et al. [44] demonstrated that Allopregnanolone administration improved T-maze and water maze task performance.

It worth noting that Allopregnanolone was approved in the united states of America for the treatment of postpartum depression [45]. Allopregnanolone is a positive modulator of GABAAR activity [5]. GABA is the primary inhibitory neurotransmitter in the brain and plays a vital role in learning and memory [46] and synaptic plasticity [47]. The hippocampus has numerous GABAA receptors that participate in molecular mechanisms of memory formation [48].

In a pilot study performed by Napoli et al. [49], allopregnanolone treatment significantly impacted GABA metabolism, oxidative stress, and other mitochondria-related outcomes. Allopregnanolone effectively lowered the content of the three markers of oxidative stress, including 2-hydroxybutyric acid [49], which is an early marker for both insulin resistance and impaired glucose regulation from increased lipid peroxidation and oxidative stress [50]. This effect may propose a primitive explanation for improving spatial memory (increased alternation score) after Allopregnanolone administration in our study.

Furthermore, Frye et al. [51] showed cognitive enhancement of progesterone (induce the formation of Allopregnanolone) that was exerted through increasing GABAA receptors activity of the hippocampus and hippocampal brain-derived neurotrophic factor (BDNF) level. BDNF the neurotrophic factor that controls synaptic plasticity [52,53]. Several studies reported increased BDNF levels following Allopregnanolone administration [54,55,56].

In Xu et al., [57] study, infusion of BDNF into the hippocampus reversed that cognitive impairment due to OA (okadaic acid) injection and upregulated Synaptophysin expression. Zhong et al. [58] also claimed the protective effect of BDNF on rat hippocampal neurons exposed to high glucose in vitro by increasing Synaptophysin levels. These studies propose a logical cause for the marked increase of relative expression of Synaptophysin in the allopregnanolone group compared to the MetS group (Table 2).

Regarding GA*P*-43, our study showed a prominent decrease in its relative expression in the allopregnanolone group compared to the MetS group (Table 3). However, there is still a significant difference between the relative expression of GA*P*-43 in the allopregnanolone group compared to the negative control group (Table 3). This result can also be explained by the ability of Allopregnanolone to increase hippocampal BDNF level [51]. It is worth noting that BDNF increases antioxidant enzymes’ activity in cells and fights against free radicals [59].

Besides, previous studies have shown that Allopregnanolone also can increase hippocampal neurogenesis [42]. Allopregnanolone increases the expression of genes that promote mitosis and reduces antimitotic gene expression [60,61]. In the present study, allopregnanolone administration, a ligand to GABAA receptors [62], caused a decremental decrease in serum glucose, serum insulin, and HOMA-IR in group IV in comparison to group III. These findings run parallel to what was previously reported by Tian et al. [63]. It has been reported that GABAARs are expressed in the primary islet β-cells [64] that regulate insulin secretion together with changes in glucose levels [65].

Another finding of our study is that the role of Allopregnanolone in improving the lipid profile indicated by the decreased levels of serum triglycerides and cholesterol. Previous studies have reported that the mechanism of Allopregnanolone action is thought to involve pregnane X receptor (PXR) activation [66]. PXR transcriptionally activates Insig-1, a protein with antilipogenic properties, which accordingly reduces the nuclear protein level of the active sterol regulatory element-binding protein 1 (SREB*P*-1) with subsequent reduction of transcription of specific lipogenic genes, causing a decrease in fatty acid, triglyceride and cholesterol synthesis [67,68].

Our findings indicate that AlloP has antihypertensive effects in experimentally induced metabolic syndrome in rats. It produced a marked decrease in systolic blood pressure (SBP), diastolic blood pressure (DBP), and mean blood pressure (MBP). These findings are in line with a previous study that found that the neurosteroid allopregnanolone effectively reduces BP and also the pressor response to stress in a mouse model of neurogenic hypertension [69]. Additionally, other clinical studies showed that high levels of Allopregnanolone were correlated with a blunted SBP response to cocaine cues in cocaine-dependent individuals [70]. The previous studies support these findings reported the antihypertensive effect of GABA-rich Chlorella in spontaneously hypertensive rats (SHR) and borderline hypertension subjects [71]. High blood pressure could be a classical feature of metabolic syndrome, and it has been reported that metabolic syndrome is present in up to 30% of hypertensive patients [72]. The sympathetic system activation is characteristic of several metabolic disorders., many of which occur more frequently in obese individuals. Obesity potentiates sympathetic system activation in patients with hypertension [73]. Multiple studies have demonstrated that metabolic syndrome is accompanied by a hyperadrenergic state independent of the presence of hypertension [74].

The elevated plasma insulin levels may account for multiple metabolic syndrome features since insulin has been demonstrated to change glucose and lipid metabolism, enhance atherogenesis, and increase blood pressure [75]. Human studies revealed a heterogenous sympathetic response with increased muscle sympathetic activity induced by insulin [76,77].

The arterial baroreceptor control of sympathetic nerve traffic undergoes a clear-cut impairment in patients with metabolic syndrome, in which both the sympathoinhibitory and the sympathoexcitatory baroreflex components are involved [74]. The BP lowering actions of Allopregnanolone involve sympatho-inhibition via upregulation of GABAA receptors that specifically contain δ subunits within the hypothalamus and amygdala [69]. These δ subunits containing GABAA receptors are presumably extrasynaptic and very sensitive to GABA and Allopregnanolone but relatively insensitive to the action of benzodiazepines [11,78].

## 5. Conclusions

The present study revealed a positive impact of allopregnanolone therapy in animal models of metabolic syndrome. Allopregnanolone therapy improved working spatial memory and biochemical markers of metabolic syndrome (serum glucose, insulin, insulin resistance, lipid profile). Moreover, it improved the expression of hippocampal Synaptophysin that may explain the improved performance in Y-maze. Further studies are recommended to evaluate other aspects of cognition with a molecular evaluation of other synaptic plasticity markers, especially BDNF and signaling pathways, to understand better the mechanism involved in allopregnanolone effects. It is advised to separate the drug’s effect from the alcohol (vehicle), so further investigations are advised with comparing MetS treated with Allopregnanolone dissolved in the vehicle with MetS treated with vehicle alone. Besides, more investigations are required to evaluate the effect of Allopregnanolone in females with metabolic syndrome considering fluctuations in female sex hormones in estrus or menstrual cycle and their effects on the brain.

## Figures and Tables

**Table 1:** The primer sequence of the studied genes.

	Primer Sequence	Accession Number
Synaptophysin	Forward primer: 5′-TCCAATCAGATGTAGTCTGGTCAG-3′ Reverse primer: 5′-AGGCCTTCTCCTGAGCTCTT-3′	NM_009305.2
GA*P*-43	Forward primer: 5′-TTTCCTCTCCTGTCCTGCTC-3′ Reverse primer: 5′-TGGACTTGGGATCTTTCCTG-′3	NM_008083.2
glyceraldehyde-3-phosphate dehydrogenase (Gapdh)	Forward primer: 5′-GGTCGGTGTGAACGGATTTGG-3′ Reverse primer: 5′-ATGTAGGCCATGAGGTCCACC-3′	NM_001289726.1

GA*P*-43: Growth Associated Protein 43.

**Table 2:** Biochemical serum and hippocampal markers.

Measured Parameters	Negative Control for Allopregnanolone	Positive Control for Allopregnanolone	MetS	MetS+ Allopregnanolone	*F*-Values	*p*-Value
Serum Glucose (mg/dL)	97.3 ± 12.9 ^a^	107.8 ± 8.2 ^a^	250.8 ± 53.8 ^b^	189.0 ± 36.6 ^c^	37.6	<0.001
Serum Insulin (µIU/L)	9.2 ± 0.9 ^a^	14.4 ± 1.1 ^b^	21.3 ± 3.9 ^c^	16.7 ± 2.0 ^b^	40.1	<0.001
HOMA-IR	2.20 ± 0.34 ^a^	3.72 ± 0.44 ^a^	12.97 ± 2.8 ^b^	7.85 ± 2.03 ^c^	33.5	<0.001
Serum Triglycerides (mg/dL)	75.5 ± 14.9 ^a^	91.2 ± 22.9 ^a^	143.4 ± 17.5 ^b^	126.7 ± 5.6 ^b^	28.7	<0.001
Serum Cholesterol (mg/dL)	130.9 ± 3.9 ^a^	146.2 ± 25.4 ^a^	204.6 ± 16.3 ^b^	153.9 ± 45.1 ^a^	11.1	<0.001
Serum HDL (mg/dL)	58.3 ± 3.4 ^a^	56.9 ± 9.1 ^a^	28.6 ± 3.2 ^b^	43.1 ± 5.1 ^c^	47.1	<0.001
Hippocampal Synaptophysin (relative expression)	1.8 ± 0.2 ^a^	1.3 ± 0.1 ^b^	0.1 ± 0.03 ^c^	0.9 ± 0.1 ^d^	155.1	<0.001
Hippocampal GAP43 (relative expression)	1.1 ± 0.03 ^a^	1.2 ± 0.3 ^a^	4.5 ± 0.6 ^b^	2.7 ± 0.5 ^c^	112.9	<0.001

There was a statistically significant difference between a variable with a different letter, no significant difference between variables with the same letter. Degree of freedom between groups equal 3 and within groups equal to 28. MetS: Metabolic syndrome; HOMA-IR: Homeostasis model assessment of insulin resistance; HDL: high-density lipoproteins; GAP 43: Growth Associated Protein 43.

**Table 3:** Arterial blood pressure measurements in the studied groups.

Measured Parameters	Negative Control for Allopregnanolone	Positive Control for Allopregnanolone	MetS	MetS+Allopregnanolone	*F*-Value	*p*-Value
Systolic BP (mmHg)	116.8 ± 4.1 ^a^	104.9 ± 4.3 ^b^	151.5 ± 11.0 ^c^	118.5 ± 5.1 ^a^	70.3	<0.001
Diastolic BP (mmHg)	84.5 ± 6.6 ^a^	86.4 ± 3.6 ^a^	113.6 ± 6.3 ^b^	90.9 ± 2.3 ^a^	56.6	<0.001
MAP (mmHg)	96.6 ± 4.2 ^a^	91.5 ± 3.7 ^a^	128.3 ± 8.7 ^b^	100.4 ± 3.4 ^a^	72.6	<0.001

There was a statistically significant difference between a variable with a different letter, no significant difference between variables with the same letter. Degree of freedom between groups equal 3 and within groups equal to 28. MAP: Mean arterial blood pressure.

**Table 4:** Correlation between alternation score in Y-maze (%) and biochemical markers.

Biochemical Parameters	Alternation Score in Y-Maze (%)	
	Correlation Coefficient (*r*)	*p* Value
Serum Glucose (mg/dL)	−0.086	0.640
Serum Insulin ((µIU/L)	−0.334	0.062
Serum Triglycerides (mg/dL)	−0.301	0.094
Serum Cholesterol (mg/dL)	−0.204	0.262
Serum HDL (mg/dL)	0.317	0.077
Hippocampal Synaptophysin (relative expression)	0.366	0.039
Hippocampal Growth Associated Protein 43 (GAP43) (relative expression)	−0.414	0.019

## Data Availability

The data that support the findings of this study are available from the corresponding author S.N.A., upon reasonable request.
